# Photocatalytic VOCs Degradation Efficiency of Polypropylene Membranes by Incorporation of TiO_2_ Nanoparticles

**DOI:** 10.3390/membranes13010050

**Published:** 2022-12-30

**Authors:** Md. Abu Hanif, Hyokyeong Shin, Danbi Chun, Hong Gun Kim, Lee Ku Kwac, Young Soon Kim

**Affiliations:** 1 Institute of Carbon Technology, Jeonju University, Jeonju 55069, Republic of Korea; 2 Graduate School of Carbon Convergence Engineering, Jeonju University, Jeonju 55069, Republic of Korea

**Keywords:** TiO_2_, polypropylene, membrane, VOC eradication, photocatalysis

## Abstract

A class of serious environmental contaminants related to air, namely volatile organic compounds (VOCs), has currently attracted global attention. The present study aims to remove harmful VOCs using as-prepared polypropylene membrane + TiO_2_ nanoparticles (PPM + TiO_2_ NPs) via the photocatalytic gas bag A method under UV light irradiation. Here, formaldehyde was used as the target VOC. The PPM + TiO_2_ NPs material was systematically characterized using various microscopic and spectroscopic techniques, including field emission scanning electron microscopy, energy-dispersive X-ray spectroscopy, X-ray diffraction, Fourier-transform infrared spectroscopy, ultraviolet–visible diffuse reflectance spectroscopy, photoluminescence spectroscopy, and contact angle measurements. These results confirm the successful preparation of PPM + TiO_2_ NPs, which can be applied to the degradation of VOCs. Photocatalytic degradation of formaldehyde gas reached 70% within 1 h of UV illumination. The energy bandgap and photoluminescence intensity reductions are responsible for the improved photocatalytic activity. These characteristics increase the charge transport while decreasing the recombination of electron–hole pairs.

## 1. Introduction

The deterioration of indoor air quality has become a major issue for scientific, economic, and social communities because it is directly related to human health. According to various studies, modern people spend almost 80% of their time indoors on average, including at home, school, shopping centers, hospitals, offices, and cars [1]. Additionally, according to the World Health Organization, air pollution caused seven million premature deaths in 2012. Among these, 4.3 million deaths were a result of indoor pollution [2].

Volatile organic compounds (VOCs) are an important class of anthropogenic/biogenic indoor air contaminants with several harmful health consequences [3]. In addition to being dangerous substances, VOCs also form harmful by-products, including secondary organic aerosols, ozone, and organic compounds [4]. The most frequently studied airborne pollutants are volatile organic compounds (VOCs) such as formaldehyde, toluene, and chloroform. However, the most typical indoor contaminant is formaldehyde, prolonged exposure to which negatively impacts human health, causing eye irritation, pneumonia, pulmonary edema, inflammation, respiratory problems, and skin irritation [5,6]. Carpets, parquets, and adhesives containing formaldehyde are used for plastic surfaces, paper products, pesticide formulations, cigarette smoke, disinfectants, household cleaning products, fabrics, water-based paints, insulating materials, particle boards, and plywood furniture made from formaldehyde-based resins, and other building materials made with urea-formaldehyde resins are the most significant indoor sources that can cause human exposure to formaldehyde [7]. The U.S. Environmental Protection Agency (EPA) and the International Agency for Research on Cancer (IARC) have designated formaldehyde as a human carcinogen and recognized human carcinogen, respectively [1,7].

Numerous air purification methods for eliminating VOCs have been suggested as solutions to this issue. Physical adsorption [8], plasma breakdown [9], catalytic oxidation [10], physical capture [11], condensation [12], and photocatalysis [13] are the primary techniques used in the prescribed remedies. In addition to having substantial potential to remove VOCs, the proposed approaches produce large quantities of hazardous intermediates, by-products, and waste. The sophisticated photocatalytic oxidation process works at ambient temperature and pressure and is both effective and clean. These advantages have led to extensive research on the potential utility of photocatalysis for water and indoor/outdoor air purification. Therefore, photocatalysis is a potential VOC elimination technology for practical applications.

Several efforts have been made to increase the removability of VOCs and bacteria in the air by introducing photo-induced oxidants into the substrate and membrane. For instance, metallic oxides with high photocatalytic activities, such as CdS, ZnO, CuO, and TiO_2_, have been introduced [14,15,16,17]. These metallic oxides can be employed to decompose VOCs in the environment. Moreover, TiO_2_ has the highest photocatalytic degradation activity and best biocompatibility and is the least poisonous. Therefore, there has been an increased interest in TiO_2_ usage. Several researchers have evaluated the efficacy of photocatalytic degradation for the gaseous phase degradation of formaldehyde, but they have employed powdered TiO_2_ or TiO_2_ supported on glass. In addition, tests have been conducted on various supporting surfaces, including stainless steel, ceramics, and polymers. An advantage of using polymeric materials is that they are less expensive as raw materials than metals and also less expensive to manufacture into finished products than glass and metals. It is to be noted that TiO_2_ can be used to eliminate contaminants by coating with various polymers [18].

A noteworthy advantage of using polypropylene as a substrate for catalytic photodegradation is that it is a polymer. Typically, the self-catalytic degradation of polypropylene involves the formation of free radicals during the initial process. In the presence of oxygen, a hydroperoxyl radical is produced following the creation of free radicals. Subsequent processes that result in the creation of hydroperoxide are initiated by the production of hydroperoxyl. Typically, the sol-gel technique, chemical vapor deposition, liquid-phase deposition, and impregnation methods are used to deposit metal/metallic particles onto or into fiber sheets [19]. However, these techniques are expensive, time-consuming, and challenging, thus restricting the production of multifunctional air filters on an industrial scale. Therefore, the development of efficient technologies for VOC treatment is urgently needed.

In recent decades, membranes have been progressively used for various purification applications, including air purification and water treatment [20]. Membrane-based approaches provide several intrinsic advantages, such as ease of synthesis, surface modifications through thin-film deposition, high degree of selectivity, and convenience of use [21]. Numerous aspects of membrane science and technology have demonstrated fast advancement, including the resistance of membranes to fouling, rates of penetration, and selectivity towards solutes. For recyclability, sample collection after treatment is a crucial step in the photocatalytic system. In the membrane separation technique, suspended photocatalysts are used to separate the photocatalysts in the medium on completion of the catalytic process. Separation systems typically use pressure-driven filtration with nanofiltration, ultrafiltration, or microfiltration membranes [22]. However, this technique causes a constant decrease in the photocatalyst concentration in an operating cycle, which results in material waste and more expensive processing. Immobilizing a photocatalyst into a membrane (photocatalyst membrane) using diverse manufacturing techniques is an innovative solution to this issue. Pollutants can accumulate on the photocatalytic membrane close to the catalyst surface, where they can be degraded [23]. In addition, membrane fouling can be reduced because the photocatalyst inclusion increases the wettability of the membrane. Photocatalytic membranes are preferred owing to their lower environmental impact, lower risk of membrane fouling, and superior capacity to oxidize and reduce pollutants. Additionally, because these two technologies (photocatalyst and membrane) operate under similar operating conditions and are simple to regulate, they can be merged. Therefore, lower operating and maintenance costs are anticipated [24].

In this study, we fabricated polypropylene membranes using TiO_2_ NPs (PPM + TiO_2_ NPs) using a simple, affordable, low-cost spray deposition method. The development of the spray deposition method is one of the most effective strategies for large-scale production. The PPM + TiO_2_ NPs product was applied to the photocatalytic VOC, which indicated formaldehyde degradation in the presence of UV irradiation and produced a remarkable result. Additionally, the photocatalytic mechanism of VOC degradation was investigated.

## 2. Materials and Methods

### 2.1. Materials

TiO_2_ 10% w/w solution (TiO_2_ NPs, dissolved in water, particle size ~31.75 ± 9.31 nm) was obtained from Sukgyung AT Co., Ltd., Ansan, Republic of Korea. A polypropylene membrane (PPM, 99.8%; MFI: 1100 g 10 min^−1^) was obtained from Sunjin Glotech, Inc., Jeonbuk, Republic of Korea. A formaldehyde solution (37%, stabilized) was obtained from Sigma-Aldrich (St. Louis, MO, USA). All materials were of analytical grade and used directly.

### 2.2. Fabrication of PPM + TiO_2_ NPs

**Figure 1:**
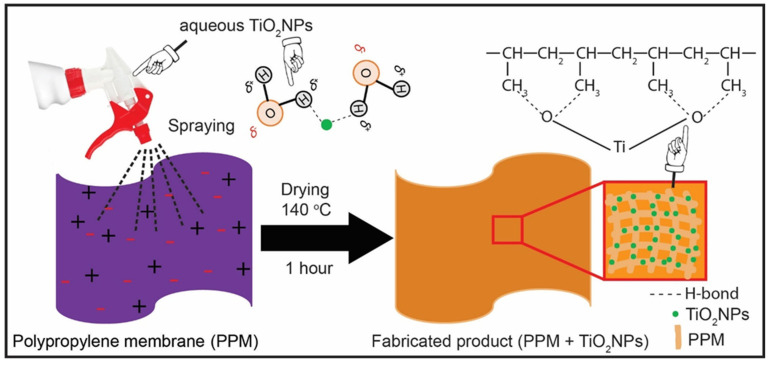
Schematic photograph for the fabrication of the PPM using TiO_2_ NPs.

PPM + TiO_2_ NPs were fabricated using a simple, facile, cost-effective spray deposition method. In a typical experiment, 10 mL of the TiO_2_ NPs solution was placed in a 20 mL beaker and sonicated for 20 min at room temperature to obtain a well-dispersed solution. Meanwhile, a PPM of 10 × 10 cm^2^ in size was measured carefully and placed on a clean, washed Petri dish. The PPM was prepared using the hydro-charging method [25]. The weight and thickness of the freshly prepared PPM were 0.3055 g and 0.23 mm, respectively. To fabricate the PPM + TiO_2_ NPs, 2 mL of a well-dispersed TiO_2_ NP solution was sprayed for the deposition of TiO_2_ NPs on the surface of the PPM. TiO_2_ NPs were deposited using a spray gun with a nozzle diameter of approximately 2 mm within a fume hood. The Petri dish with samples was positioned around 10 cm away from the nozzle. Then, the fabricated PPM was dried at room temperature and further heated at 140 °C in an oven for 1 h to completely remove water molecules. Thus, we fabricated several pieces of membranes that were almost homogeneous. The average weight and thickness of the as-prepared PPM + TiO_2_ NPs were 0.3736 ± 0.0034 g and 0.25 mm, respectively. Therefore, approximately 0.0681 ± 0.0034 g of TiO_2_ was deposited on the PPM surface. Figure 1 shows a schematic diagram of the fabrication of the PPM using TiO_2_ NPs.

### 2.3. Characterization

Field emission scanning electron microscopy (FE-SEM), energy-dispersive X-ray spectroscopy (EDS), and transmission electron microscopy (TEM) were used to study the morphological characteristics of the fabricated materials. To examine the crystal structures of the compounds, X-ray diffraction (XRD) was applied. The diffractometer (X’Pert PRO, PANalytical, Lelyweg, Almelo, Netherlands) was equipped with a Cu Kα (*λ* = 1.5406 Å) radiation source. The chemical state, oxidation state, and binding energies of the samples were examined using X-ray photoelectron spectroscopy (XPS, NEXSA, Thermo Fisher Scientific, East Grinstead, West Sussex, UK). Fourier-transform infrared (FTIR, Thermo Fisher Scientific, Nicolet iS5) spectroscopy was used to investigate the functional groups of the materials. The optical characteristics and bandgaps of the materials were evaluated using a UV–Vis spectrophotometer (Perkin Elmer Lambda 25, Ayer Rajah, Singapore). The photoluminescence (PL) spectra were measured using a spectrophotometer (FP-6500, Jasco). Thermal and mechanical analyses, including thermogravimetric analysis (TGA, SDT Q600), differential scanning calorimetry (DSC, Q20), thermomechanical analysis (TMA, Q400), and dynamic mechanical analysis (DMA, Q800), were performed using a TA instrument (Universal V4.5 A TA Instruments, New Castle, DE, USA). The water contact angle was measured using a Phoenix 300 Touch analyzer (SEO Co., Ltd., Pyeongtaek, Republic of Korea).

### 2.4. Measurements of Photocatalytic Performance

The photocatalytic VOC decomposition test was performed by the Korea Institute of Ceramic Engineering and Technology (KICET), which issued an accurate procedure for the experimental results and a reliable accredited certificate. The test method was presented as gas bag A by the Korea Photocatalyst Association (Association of Korea Photocatalysts, AKP). This is a method to immediately determine the decomposition performance by irradiating a 5 L gas bag filled with VOCs and comparing the concentration with the unirradiated sample.

A photocatalytic VOC degradation test was performed using formaldehyde as the model VOC. In a typical experiment, the test gas formaldehyde was injected into a Teflon gas bag connected to a gas valve containing the PPM and PPM + TiO_2_ NPs. Ultraviolet rays were irradiated to determine the degree of decomposition of the test gas. Formaldehyde gas was injected, resulting in a formaldehyde concentration of 100 ppm. Formaldehyde degradation in the gas bag was assessed to evaluate the performance of the PPM and PPM + TiO_2_ NP membranes with a size of 10 × 10 cm^2^. In this study, a GASTEC Detector Tube No. 91M with a Gastec pump (GV-100S) was used to measure the formaldehyde concentration. The experiments were performed using a UV light source from a Sankyo Denki lamp FL20SBLB (1 mW/cm^2^). The samples were pretreated with UV light for 16 h to remove any impurities and were kept in the dark for 2 h to evaluate adsorption. Thereafter, to measure only the photocatalytic decomposition performance, excluding the adsorption performance, the gas bag was filled with formaldehyde gas to maintain the exact concentration (100 ppm) before the application of the light source. The formaldehyde gas bag was quickly placed in a shading box for 2 h under dark conditions and at room temperature. The gas concentration was measured twice using a detection tube, and the average value was obtained as the concentration under dark conditions. Finally, the gas concentration was measured for both conditions (dark and light) from the indicator tube with the naked eye.

## 3. Results

### 3.1. Morphological Investigation

**Figure 2:**
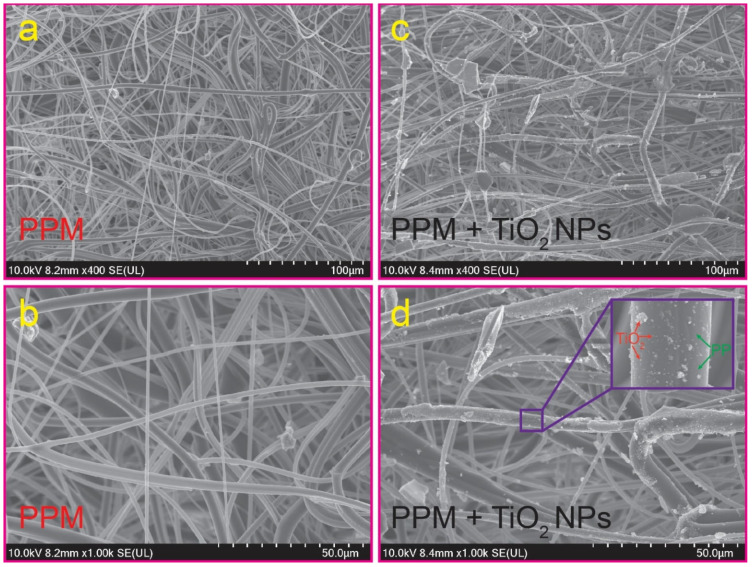
FE-SEM photographs of the PPM (**a**,**b**) and PPM + TiO_2_ NPs (**c**,**d**).

FE-SEM was used to examine the morphology of the PPM and PPM + TiO_2_ NPs, as shown in Figure 2. Interconnected open pore architectures with randomly oriented microfibers were visible in both the PPM and PPM + TiO_2_ NP compounds. Figure 2a,b show that the PPM possessed uniform fibers with a diameter range of 1.07 to 4.82 µm. The average diameter of the PPM was 2.43 ± 1.18 nm. However, Figure 2c,d reveal that after combining the TiO_2_ NPs and the PPM, there was no discernible change in the average diameter. However, it is clearly seen that the nanosized spherical aggregated TiO_2_ NPs (red arrows, inset Figure 2d) successfully deposited onto the smooth surface of the PPM (green arrows, inset Figure 2d) and were tightly connected to each other.

**Figure 3:**
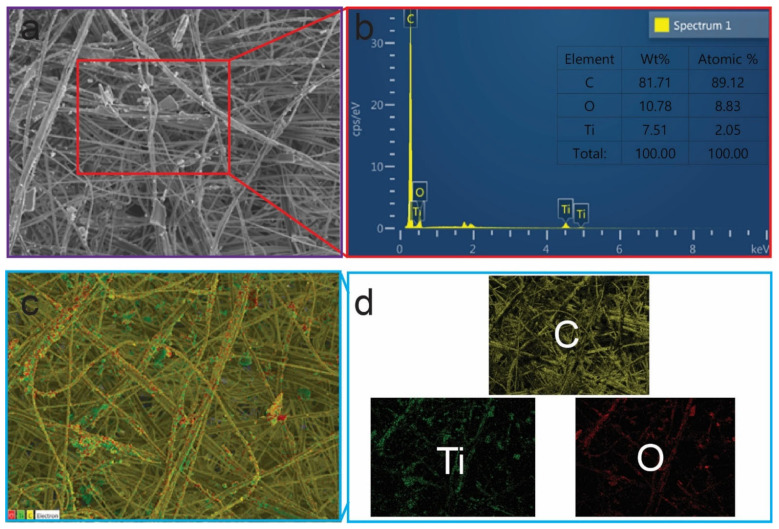
EDS (**a**,**b**) and elemental mapping (**c**,**d**) analysis of PPM + TiO_2_ NPs.

Furthermore, a better understanding of the distribution and identity of elements in the as-prepared PPM + TiO_2_ NPs was determined using EDS and elemental mapping analysis (Figure 3). The presence of three representative elements (C, Ti, and O) in the PPM + TiO_2_ NPs and the absence of any other elements indicate that the PPM + TiO_2_ NPs were created by the combination of only PPM and TiO_2_ NPs. Additionally, the relative weights and atomic percentages of the constituents demonstrate that the TiO_2_ NPs were successfully deposited onto the surface of the PPM (Figure 3a,b). The elemental mapping results show that the three elements were homogeneously distributed in the PPM + TiO_2_ NPs system (Figure 3c,d).

**Figure 4:**
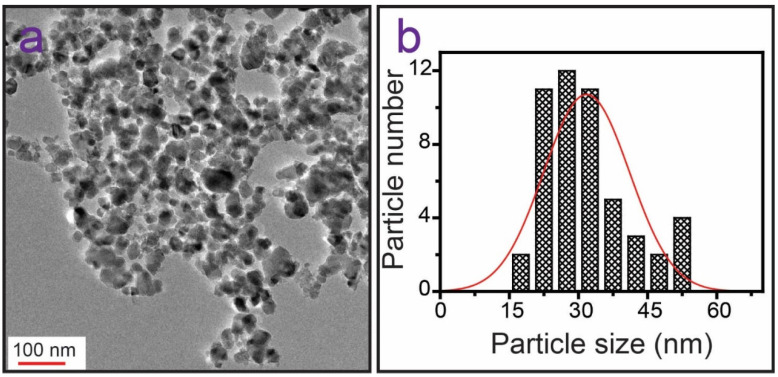
(**a**) TEM image of TiO_2_ NPs. (**b**) Particle size distribution graph of TiO_2_ NPs (50 particles were counted).

In addition, TEM was used to investigate the morphology and size of the TiO_2_ NPs. Figure 4a shows a TEM image of the TiO_2_ NPs, which indicates that the TiO_2_ NPs were approximately spherical, which supports the FE-SEM findings. Figure 4b shows the particle size distribution (50 particles were counted). According to the size distribution, the TiO_2_ NPs ranged from 23 to 41 nm, with an average size of 31.75 ± 9.31 nm.

### 3.2. Structural Investigation

**Figure 5:**
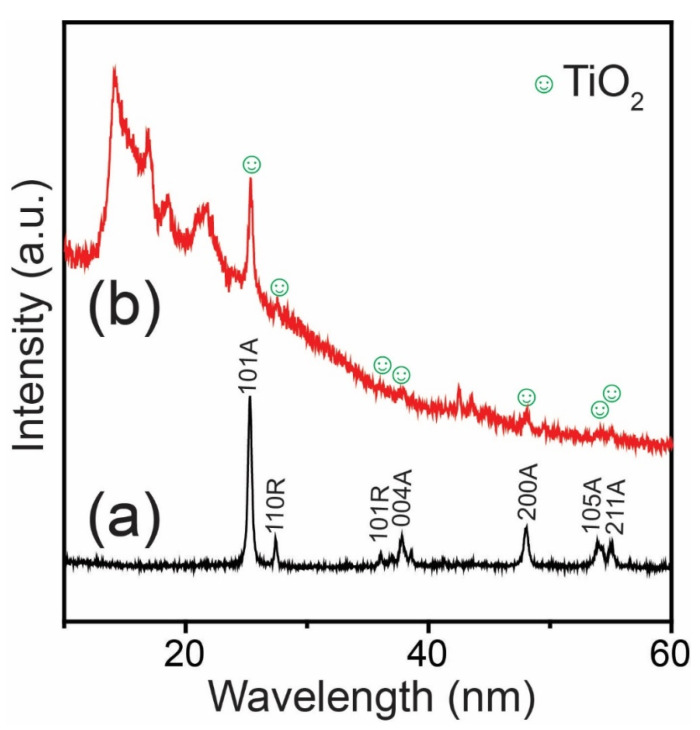
XRD spectra of (**a**) TiO_2_ NPs and (**b**) PPM + TiO_2_ NPs.

XRD plots of the TiO_2_ NPs and PPM + TiO_2_ NPs are shown in Figure 5. The TiO_2_ NPs show two types of XRD phases (Figure 5a). The diffraction peaks at 2θ angles of 25.28°, 37.90°, 47.77°, 53.85°, and 55.08° are attributed to the anatase phases of the (101), (004), (200), (105), and (211) planes, respectively [26]. In contrast, the peaks at 27.43° and 36.06° are responsible for the rutile phases of (110) and (101) planes, respectively [27]. As shown in Figure 5b, all the representative peaks of TiO_2_ were observed in the PPM + TiO_2_ NPs sample. Moreover, the diffraction peaks at 2θ angles of 14.13°, 16.90°, 18.48°, and 21.63° correspond to the α-forms (PPM) of the (110), (040), (130), and (060) crystal planes, respectively. There was no obvious difference in the diffraction angles with TiO_2_ NPs’ incorporation. Therefore, it was concluded that adding TiO_2_ NPs did not alter the polypropylene matrix’s crystal structure.

**Figure 6:**
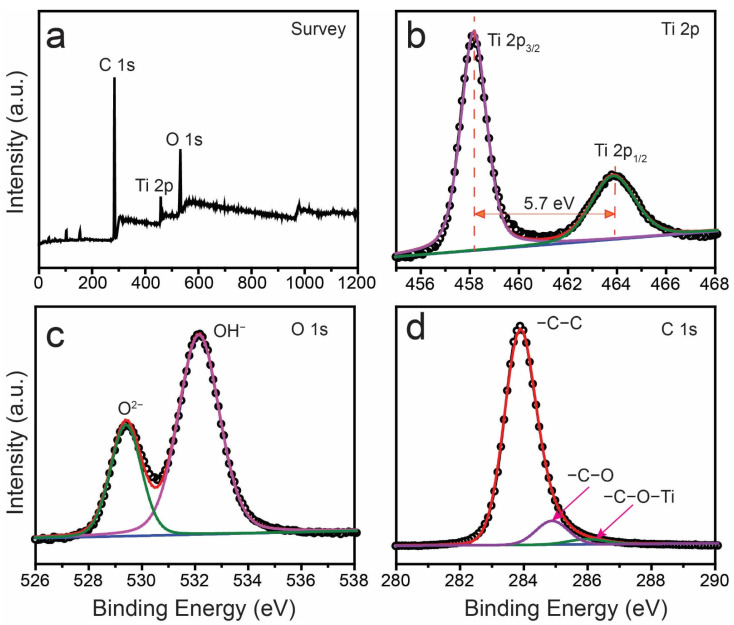
XPS spectra of PPM + TiO_2_ NPs: (**a**) survey, (**b**) Ti 2p, (**c**) O 1s, and (**d**) C 1s.

XPS spectra were used to analyze the qualitative data regarding the chemical identity, oxidation state, and binding energy of the as-prepared PPM + TiO_2_ NPs, as shown in Figure 6. Figure 6a shows the XPS survey spectrum of the PPM + TiO_2_ NPs. The survey spectrum demonstrates that three characteristic elements (C, Ti, and O) were present in the PPM + TiO_2_ NPs. These results are consistent with the EDS analysis results. Carbon came mainly from the PPM, and Ti and O from the TiO_2_ NPs. There were no other constituents, proving that the PPM and the TiO_2_ NPs were combined successfully to generate PPM + TiO_2_ NPs. High-resolution Ti 2p peaks are shown in Figure 6b. The two peaks, Ti 2p_3/2_ and Ti 2p_1/2_, correspond to binding energies of 458.24 and 463.94 eV, respectively. The distance 5.7 eV between the two Ti 2p peaks indicates that all of the Ti in the PPM + TiO_2_ NPs sample is in the valence state of Ti^4+^ [28]. Two deconvoluted O 1s peaks are shown in Figure 6c. The lower binding energy peak at 529.25 eV is assigned to Ti–O bonds in the O^2−^ chemical state of the TiO_2_ lattice. In contrast, the higher binding energy peaks at 532.16 eV are ascribed to the surface OH^−^ groups [29]. The OH^−^ group was crucial for the photocatalytic process. It is possible for OH^−^ to spread across the surface of the TiO_2_ compartment and be transformed into highly reactive •OH^−^, which promotes the oxidative destruction of contaminants. Additionally, OH^−^ facilitates the transfer of the generated hole and improves the separation effectiveness of the generated electron–hole pair [30]. The three deconvoluted C peaks located at the binding energies of 283.88, 284.95, and 285.97 eV were attributed to –C–C, –C–O, and –C–O–Ti bonds, respectively (Figure 6d) [26,31].

**Figure 7:**
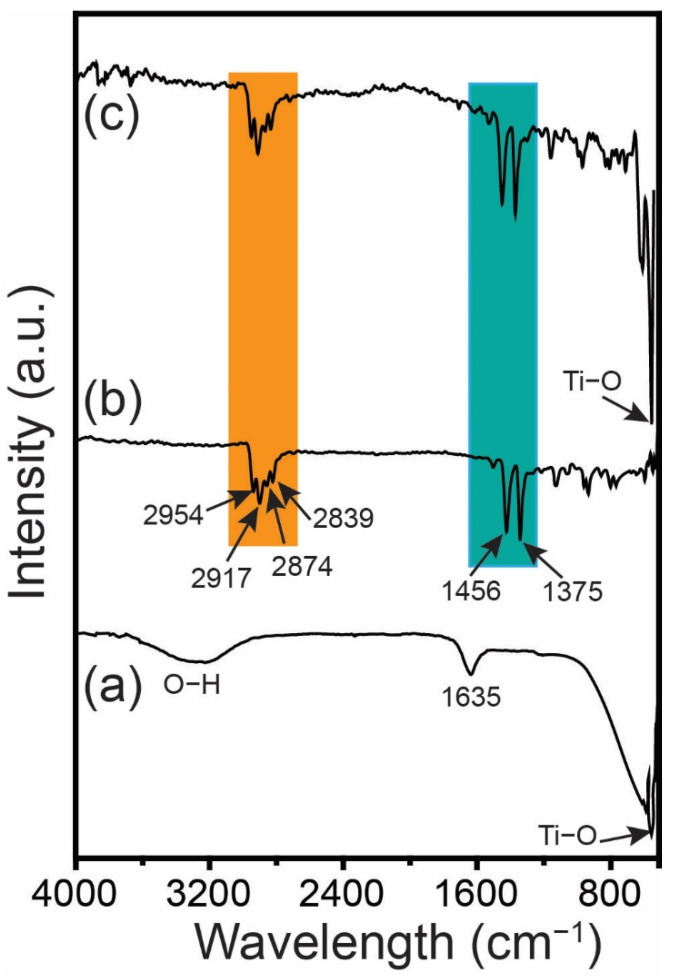
FTIR spectra of (**a**) TiO_2_ NPs, (**b**) PPM, and (**c**) PPM + TiO_2_ NPs.

Furthermore, FTIR spectroscopy was used to investigate the functional groups and bonding properties of the TiO_2_ NPs, the PPM, and the PPM + TiO_2_ NPs (Figure 7). The TiO_2_ NPs showed a peak at approximately 556 cm^−1^ because of the Ti–O vibrations stretching mode (Figure 7a). Additionally, the peaks observed at 1635 cm^−1^ and a broad peak at 3200–3500 cm^−1^ correspond to the bending and stretching vibrations of O–H, respectively, from surface-adsorbed water molecules [26,27]. In Figure 7b, the PPM peaks at 1456 and 1375 cm^−1^ are associated with the bending vibration of –CH_2_ and the symmetric deformation of –CH_3_, respectively. In addition, peaks for –CH_3_ and –CH_2_ symmetric stretching vibrations are observed at 2874 and 2839 cm^−1^, respectively. The –CH_3_ and –CH_2_ asymmetric stretching vibrations are attributed to the peaks at 2954 and 2917 cm^−1^, respectively [32]. All representative peaks of TiO_2_ NPs and PPM are found in the spectrum of the PPM + TiO_2_ NPs sample, confirming the successful preparation of PPM + TiO_2_ NPs. It can be concluded that the bonding environment of the PPM matrix was unaffected by the addition of TiO_2_ NPs. These results are consistent with the XRD results.

### 3.3. Optical Investigation

**Figure 8:**
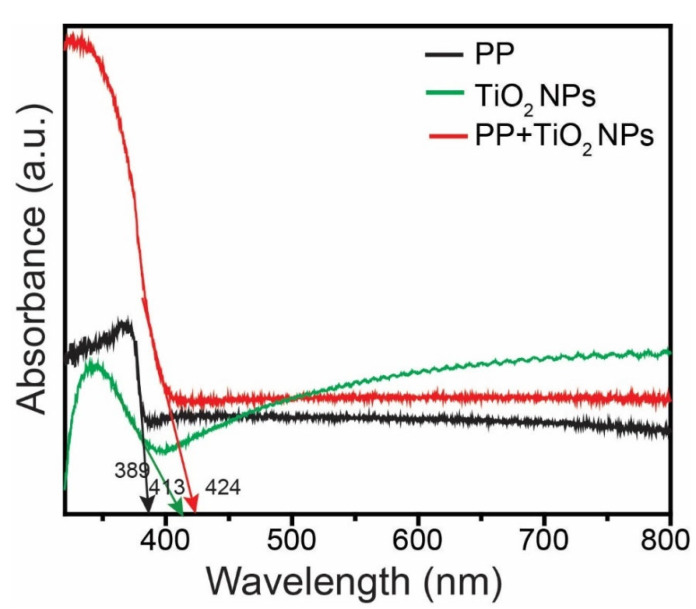
UV–Vis DRS spectra of PPM, TiO_2_ NPs, and PPM + TiO_2_ NPs.

UV–Vis and photoluminescence (PL) spectroscopy were used to examine the optical characteristics of the PPM, TiO_2_ NPs, and PPM + TiO_2_ NPs. The absorption spectra of these compounds are shown in Figure 8. The absorption edges of the PPM, TiO_2_ NPs, and PPM + TiO_2_ NPs are 389, 413, and 424 nm, respectively. These findings demonstrate that the absorption edge moved closer to the visible range when the TiO_2_ NPs and PPM were combined. Additionally, the bandgap energy (E_g_) of the PPM, TiO_2_ NPs, and PPM + TiO_2_ NPs were calculated from the UV–Vis absorption data using Planck’s energy Equation (1):

E_g_ = hc/λ = (1240/λ) eV (1)

where h denotes Planck’s constant, c refers to the velocity of light, and λ is the wavelength of the light. The calculated E_g_ values of the PPM, TiO_2_ NPs, and PPM + TiO_2_ NPs were 3.19, 3.00, and 2.92 eV, respectively. Moreover, after the combination of the PPM and TiO_2_ NPs, the E_g_ value decreased significantly compared to that of the PPM and TiO_2_ NPs alone. The production of electron–hole pairs was accelerated, and the recombination rate was decreased by the lower E_g_ value. The declining E_g_ value led to an increase in the photocatalytic efficiency of the semiconductor materials [33]. Therefore, when used as a photocatalyst material, the PPM + TiO_2_ NPs combination functions in both UV and visible light.

**Figure 9:**
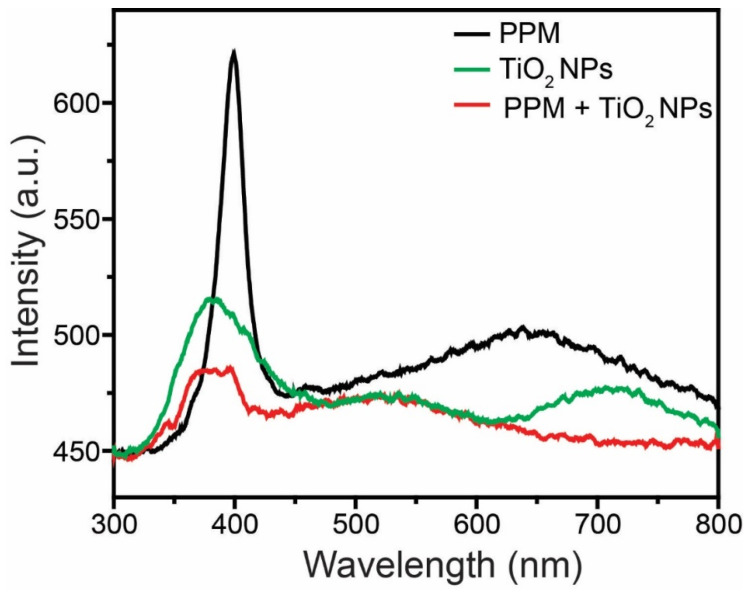
PL spectra of PP membrane, TiO_2_ NPs, and PPM + TiO_2_ NPs.

The recombination rates of the PPM, TiO_2_ NPs, and PPM + TiO_2_ NPs were evaluated by PL analysis. The PL plots of the PPM, TiO_2_ NPs, and PPM + TiO_2_ NPs samples were collected over a scanning range from 300 to 800 nm (Figure 9). The peaks of maximum intensity in the plots of the PPM, TiO_2_ NPs, and PPM + TiO_2_ NPs samples are observed at a λ_max_ of approximately 381–398 nm owing to the band–band PL phenomenon. Furthermore, because of the TiO_2_ NPs on the surface of the PPM in the PPM + TiO_2_ NPs, the emission intensity in the PL spectra of the PPM + TiO_2_ NPs was considerably reduced compared to that of the PPM and TiO_2_ NPs. This result indicates that the recombination of the photo-induced charge carriers in the PPM + TiO_2_ NPs is lower than that in the others. The diminished electron–hole pair recombination increases the separation and transfer of the photoexcited charge carriers, which boosts the activity of the product [34].

### 3.4. Thermal and Mechanical Properties Investigation

**Figure 10:**
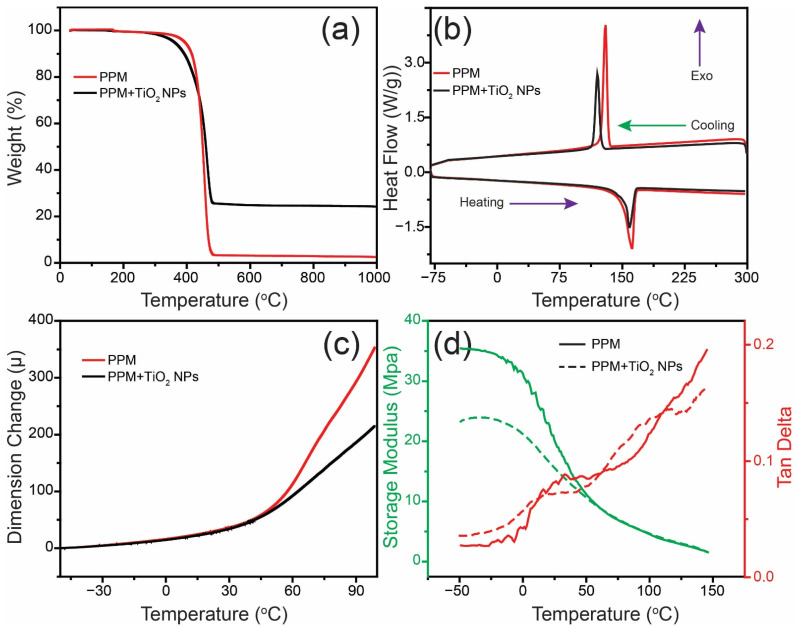
Thermal properties of PPM and PPM + TiO_2_ NPs: (**a**) TGA, (**b**) DSC, (**c**) TMA, and (**d**) DMA.

The thermal and mechanical properties of the PPM and the PPM + TiO_2_ NPs were analyzed using TA instruments, including TGA, DSC, TMA, and DMA, under a nitrogen (N_2_) atmosphere. Figure 10a shows the TGA curves of the samples. After thermal examination at temperatures up to 1000 °C, both samples thermally decomposed at different stages. Thermal dehydration of the PPM and the PPM + TiO_2_ NPs caused an initial weight loss from room temperature to 165 °C. According to the theoretical value, two water molecules per unit of the formula were lost during thermal hydration [35]. The samples showed a second weight loss after heating to 256 °C and a final weight loss of almost 475 °C. Consequently, the organic components of the PPM and the PPM + TiO_2_ NPs were broken down. Further heating to 1000 °C did not result in any additional weight loss. Figure 10b shows the DSC plots of the PPM and the PPM + TiO_2_ NPs. It can be concluded that two thermal reactions occurred within this period: one was endothermic, and the other was exothermic. Endothermic peaks were observed during the heating process at 161.56 and 158.45 °C for the PPM and the PPM + TiO_2_ NPs, respectively. On the other hand, the exothermic peaks were observed during the cooling process at 129.73 and 120.23 °C for the PPM and the PPM + TiO_2_ NPs, respectively. It can be concluded that both products are stable because no further thermal peaks were observed in these curves. Figure 10c,d display the TMA and DAM experimental data, respectively. The results show a negligible difference between the PPM and the PPM + TiO_2_ NPs. The thermomechanical properties remained almost unchanged after TiO_2_ NP addition to the PPM surface. A detailed investigation is further necessary to gain a better understanding.

### 3.5. Photocatalytic VOC degradation

The photocatalytic VOC degradation efficiencies of the PPM and PPM + TiO_2_ NPs samples were examined under UV illumination. Table 1 shows the photocatalytic degradation of formaldehyde under both dark (adsorption) and UV light (photocatalyst) conditions for the PPM and the PPM + TiO_2_ NPs. The results demonstrate that no gas was detected during the adsorption process, indicating that 100% of the gas remained in the gas bag during the photocatalytic experiment. Therefore, the gas bag was maintained at a concentration of 100 ppm for formaldehyde, and the photocatalytic performances of the PPM and the PPM + TiO_2_ NPs were inspected at room temperature for 1 h of UV irradiation. The tested gas concentrations were measured after 1 h of UV irradiation. The experiments were performed twice under identical conditions to evaluate the accuracy of the results. The average values are presented in Table 1. The photocatalytic results indicate that after 1 h of UV irradiation, there was no noticeable change in formaldehyde concentration by using the PPM. However, on using PPM + TiO_2_ NPs, 30 ppm gas remained in the gas bag, which confirms that 70% of the formaldehyde was degraded by the assistance of UV light for 1 h. Therefore, TiO_2_ NPs play a crucial role in enhancing the VOC degradation activity of the PPM. The improved photocatalytic activity is attributed to several factors, including a narrow bandgap and reduced photoluminescence intensity. After TiO_2_ NPs’ addition to the PPM surface, the bandgap of the PPM + TiO_2_ NPs was much smaller than that of the PPM and TiO_2_ compounds separately, enabling the use of broad-wavelength light in the spectrum. In addition, the lowered photoluminescence intensity of the PPM + TiO_2_ NPs overcomes the electron–hole pair recombination effect. These factors increase the number of photoexcited electrons and the flow of electron–hole pairs, thus improving the VOC photodegradation performance of the PPM + TiO_2_ NPs.

### 3.6. Photocatalytic VOC Degradation Mechanism

**Figure 11:**
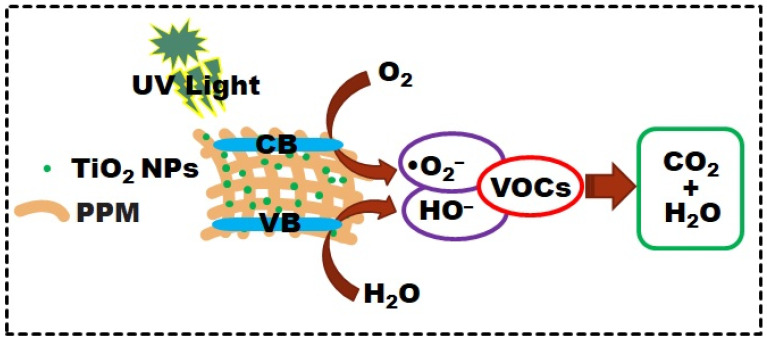
Schematic for the photocatalytic VOC degradation mechanism.

The photocatalytic VOC degradation mechanism using the as-prepared PPM + TiO_2_ NPs is shown in Figure 11. When the PPM + TiO_2_ NP membrane is in contact with a UV light source, it is excited and generates electron–hole pairs. Hole storage is performed in the valence band (VB), and electron holding is maintained in the conduction band (CB). Electrons serve as reducing agents, whereas holes act as oxidizing agents. OH^−^ is produced when water molecules interact with VB holes to produce •OH free radicals. Conversely, •O_2^−^_ is created when dissolved oxygen molecules react with photogenerated electrons in the CB. Furthermore, •O_2^−^_ free radicals produce •OH free radicals. In the photocatalytic degradation of VOCs, the produced reactive oxygen species (ROS; •O_2^−^_, HO•) are crucial elements. VOCs engage in an oxidation reaction with ROS to degrade into CO_2_ and H_2_O and also produce some non-hazardous products. A summary of the photocatalytic VOC degradation process is shown in Equation (2).

(•O_2^−^_, HO•) + VOCs → CO_2_ + H_2_O (2)

### 3.7. Wettability Investigation

**Figure 12:**
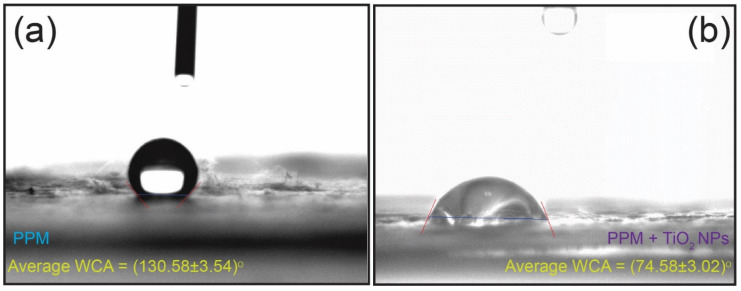
Pictorial representation for the water contact angle of the (**a**) PPM and (**b**) PPM + TiO_2_ NPs.

Wettability is a vital factor for the photocatalytic degradation of water-soluble organic pollutants when membrane-type samples are used. To determine the wettability of the PPM + TiO_2_ NPs sample, we performed a water contact angle (WCA) experiment. In addition, the PPM was used in the control experiment. Figure 12 shows the WCA results for the PPM and the PPM + TiO_2_ NPs. We performed the WCA experiment five times and calculated the average WCA for both samples (Table 2). The average WCA of the PPM was 130.58 ± 3.54° (Figure 12a), indicating that the PPM sample is hydrophobic in nature. In contrast, the average WCA of the PPM + TiO_2_ NPs sample was 74.58 ± 3.02° (Figure 12b), which confirms that the PPM + TiO_2_ NPs sample is hydrophilic in nature. Hydrophilic materials frequently react with water. Hence, water can easily penetrate the interior of the membrane owing to its high hydrophilicity, and this hydrophilicity provides superior photocatalytic activity for the breakdown of water-soluble organic pollutants. According to the WCA experiment, the PPM + TiO_2_ Ns membrane is more hydrophilic than the PPM. Therefore, a more hydrophilic surface containing the PPM + TiO_2_ NP membrane is advantageous for improving the photocatalytic performance in the degradation of water-soluble organic pollutants.

## 4. Conclusions

We fabricated a PPM using TiO_2_ NPs through a straightforward, cost-effective, and hassle-free spray deposition technique and applied it as a VOC-elimination catalyst using the gas bag A method. The as-prepared PPM + TiO_2_ NP product was systematically characterized using various advanced instruments. In the PPM + TiO_2_ NP sample, the PPM and the TiO_2_ NPs were connected by interconnecting open pore architectures with randomly oriented microfibers, as observed via FE-SEM. The XRD and FTIR measurements demonstrated that the addition of TiO_2_ NPs to the PPM surface did not change the crystal nature of the PPM. Furthermore, the XPS results showed that the product was successfully prepared without any impurities. Moreover, the UV–Vis data confirmed that the PPM + TiO_2_ NPs functioned as a suitable photocatalyst. According to the PL spectroscopy, the PPM + TiO_2_ NP material had the lowest electron–hole pair recombination effect compared to PPM and TiO_2_ NPs separately. The as-synthesized PPM + TiO_2_ NPs were used as photocatalysts for removing hazardous formaldehyde gas under UV illumination. After 1 h of UV irradiation, 70% of the formaldehyde gas was effectively removed using the PPM + TiO_2_ NPs. This enhanced photocatalytic efficiency was due to the decline in the E_g_ value and PL intensity, which reduced the recombination of photogenerated electron–hole pairs and boosted the electron flow rate. Additionally, PPM + TiO_2_ NPs can be used for the degradation of water-soluble toxic organic pollutants owing to their moderate hydrophilicity. The present study not only focuses on the degradation of VOCs but also encourages further research on the degradation of water-soluble toxic organic pollutants and bacteria. Therefore, our catalyst can be used for practical applications in air purification and wastewater treatment processes.

## Figures and Tables

**Table 1:** Photocatalytic VOC degradation using the PPM + TiO_2_ NP membrane.

Sample Name	Test Gas	Condition	Concentration (ppm)		Results
			Initial	After 1 h	
PPM	Formaldehyde	Dark	100	100	0%
		UV light	100	100	0%
PPM + TiO_2_ NPs	Formaldehyde	Dark	100	100	0%
		UV light	100	30	70%

**Table 2:** Water contact angle of the PPM and PPM + TiO_2_ NPs in different runs.

Serial No.	Sample Name	
	PPM	PPM + TiO_2_ NPs
1st run	133	75.87
2nd run	128.47	76.52
3rd run	125.46	76.38
4th run	135.7	75.57
5th run	130.25	68.57
Average	130.58	74.58

## Data Availability

Not applicable.

## References

[1] Tasbihi M., Bendyna J.K., Notten P.H.L., Hintzen H.T. (2015). A Short Review on Photocatalytic Degradation of Formaldehyde. J. Nanosci. Nanotechnol..

[2] World Health Organization (2014). Public Health, Environmental and Social Determinants of Health (PHE).

[3] Yu B.F., Hu Z.B., Liu M., Yang H.L., Kong Q.X., Liu Y.H. (2009). Review of research on air-conditioning systems and indoor air quality control for human health. Int. J. Refrig..

[4] Cheng Y.-H., Lin C.-C., Hsu S.-C. (2015). Comparison of conventional and green building materials in respect of VOC emissions and ozone impact on secondary carbonyl emissions. Build. Environ..

[5] Li W., Liang R., Hu A., Huang Z., Zhou Y.N. (2014). Generation of Oxygen Vacancies in Visible Light Activated One-Dimensional Iodine TiO_2_ Photocatalysts. RSC Adv..

[6] Lin L., Chai Y., Zhao B., Wei W., He D., He B., Tang Q. (2013). Photocatalytic Oxidation for Degradation of VOCs. Open J. Inorg. Chem..

[7] Sarigiannis D.A., Karakitsios S.P., Gotti A., Liakos I.L., Katsoyiannis A. (2011). Exposure to major volatile organic compounds and carbonyls in European indoor environments and associated health risk. Environ. Int..

[8] Lee K.J., Shiratori N., Lee G.H., Miyawaki J., Mochida I., Yoon S.-H., Jang J. (2010). Activated carbon nanofiber produced from electrospun polyacrylonitrile nanofiber as a highly efficient formaldehyde adsorbent. Carbon.

[9] Zhu X., Gao X., Qin R., Zeng Y., Qu R., Zheng C., Tu X. (2015). Plasma-catalytic removal of formaldehyde over Cu–Ce catalysts in a dielectric barrier discharge reactor. Appl. Catal. B Environ..

[10] Zhang J., Li Y., Wang L., Zhang C., He H. (2015). Catalytic oxidation of formaldehyde over manganese oxides with different crystal structures. Catal. Sci. Technol..

[11] Zhao X., Li Y., Hua T., Jiang P., Yin X., Yu J., Ding B. (2017). Cleanable Air Filter Transferring Moisture and Effectively Capturing PM_2.5_. Small.

[12] Wang M., Lawal A., Stephenson P., Sidders J., Ramshaw C. (2011). Post-combustion CO_2_ capture with chemical absorption: A state-of-the-art review. Chem. Eng. Res. Des..

[13] Abidi M., Assadi A.A., Bouzaza A., Hajjaji A., Bessais B., Rtimi S. (2019). Photocatalytic indoor/outdoor air treatment and bacterial inactivation on CuxO/TiO_2_ prepared by HiPIMS on polyester cloth under low intensity visible light. Appl. Catal. B Environ..

[14] Ouyang W., Liu S., Yao K., Zhao L., Cao L., Jiang S., Hou H. (2018). Ultrafine hollow TiO_2_ nanofibers from core-shell composite fibers and their photocatalytic properties. Compos. Commun..

[15] Harish S., Archana J., Sabarinathan M., Navaneethan M., Nisha K.D., Ponnusamy S., Muthamizhchelvan C., Ikeda H., Aswal D.K., Hayakawa Y. (2017). Controlled structural and compositional characteristic of visible light active ZnO/CuO photocatalyst for the degradation of organic pollutant. Appl. Surf. Sci..

[16] Lv D., Wang R., Tang G., Mou Z., Lei J., Han J., De Smedt S., Xiong R., Huang C. (2019). Ecofriendly Electrospun Membranes Loaded with Visible-Light-Responding Nanoparticles for Multifunctional Usages: Highly Efficient Air Filtration, Dye Scavenging, and Bactericidal Activity. ACS Appl. Mater. Interfaces.

[17] Li Q., Li X., Wageh S., Al-Ghamdi A.A., Yu J. (2015). CdS/Graphene Nanocomposite Photocatalysts. Adv. Energy Mater..

[18] Curcio M.S., Oliveira M.P., Waldman W.R., Sánchez B., Canela M.C. (2015). TiO_2_ sol-gel for formaldehyde photodegradation using polymeric support: Photocatalysis efficiency versus material stability. Environ. Sci. Pollut. Res..

[19] Park S., Park J., Heo J., Hong B.Y., Hong J. (2017). Growth behaviors and biocidal properties of titanium dioxide films depending on nucleation duration in liquid phase deposition. Appl. Surf. Sci..

[20] Das R., Sarkar S., Chakraborty S., Choi H., Bhattacharjee C. (2014). Remediation of antiseptic components in wastewater by photocatalysis using TiO_2_ nanoparticles. Ind. Eng. Chem. Res..

[21] Fane A.G., Wang R., Hu M.X. (2015). Synthetic membranes for water purification: Status and future. Angew. Chem. Int. Ed..

[22] Darowna D., Wróbel R., Morawski A.W., Mozia S. (2017). The influence of feed composition on fouling and stability of a polyethersulfone ultrafiltration membrane in a photocatalytic membrane reactor. Chem. Eng. J..

[23] Starr B.J., Tarabara V.V., Herrera-Robledo M., Zhou M., Roualdès S., Ayral A. (2016). Coating porous membranes with a photocatalyst: Comparison of LbL self-assembly and plasma-enhanced CVD techniques. J. Memb. Sci..

[24] Iglesias O., Rivero M.J., Urtiaga A.M., Ortiz I. (2016). Membrane-based photocatalytic systems for process intensification. Chem. Eng. J..

[25] Kyung B.I., Young K.H. (2014). Development of a Melt-blown Nonwoven Filter for Medical Masks by Hydro Charging. Text. Sci. Eng..

[26] Akter J., Hanif M.A., Islam M.A., Sapkota K.P., Hahn J.R. (2021). Selective growth of Ti^3+^/TiO_2_/CNT and Ti^3+^/TiO_2_/C nanocomposite for enhanced visible-light utilization to degrade organic pollutants by lowering TiO_2_-bandgap. Sci. Rep..

[27] Lee C.H., Rhee S.W., Choi H.W. (2012). Preparation of TiO_2_ nanotube/nanoparticle composite particles and their applications in dye-sensitized solar cells. Nanoscale Res. Lett..

[28] Wierzbicka E., Schultz T., Syrek K., Sulka G.D., Koch N., Pinna N. (2022). Ultra-stable self standing Au nanowires/TiO_2_ nanoporous membrane system for high-performance photoelectrochemical water splitting cells. Mater. Horiz..

[29] Zhu X., Wen G., Liu H., Han S., Chen S., Kong Q., Feng W. (2019). One-step hydrothermal synthesis and characterization of Cu-doped TiO_2_ nanoparticles/nanobucks/nanorods with enhanced photocatalytic performance under simulated solar light. J. Mater. Sci. Mater. Electron..

[30] Tian M.-J., Liao F., Ke Q.-F., Guo Y.-J., Guo Y.-P. (2017). Synergetic effect of titanium dioxide ultralong nanofibers and activated carbon fibers on adsorption and photodegradation of toluene. Chem. Eng. J..

[31] Zhu X., Dai Z., Xu K., Zhao Y., Ke Q. (2019). Fabrication of Multifunctional Filters via Online Incorporating Nano-TiO_2_ into Spun-Bonded/Melt-Blown Nonwovens for Air Filtration and Toluene Degradation. Macromol. Mater. Eng..

[32] Sun F., Li T.-T., Ren H., Jiang Q., Peng H.-K., Lin Q., Lou C.-W., Lin J.-H. (2019). PP/TiO_2_ Melt-Blown Membranes for Oil/Water Separation and Photocatalysis: Manufacturing Techniques and Property Evaluations. Polymers.

[33] Hanif M.A., Kim Y.S., Ameen S., Kim H.G., Kwac L.K. (2022). Boosting the Visible Light Photocatalytic Activity of ZnO through the Incorporation of N-Doped for Wastewater Treatment. Coatings.

[34] Hanif M.A., Akter J., Islam M.A., Sapkota K.P., Hahn J.R. (2021). Visible-light-driven enhanced photocatalytic performance using cadmium-doping of tungsten (VI) oxide and nanocomposite formation with graphitic carbon nitride disks. Appl. Surf. Sci..

[35] Hanif M.A., Lee I., Akter J., Islam M.A., Zahid A.A.S.M., Sapkota K.P., Hahn J.R. (2019). Enhanced photocatalytic and antibacterial performance of ZnO nanoparticles prepared by an efficient thermolysis method. Catalysts.

